# Effects of cisplatin on different haemopoietic progenitor cells in mice.

**DOI:** 10.1038/bjc.1982.216

**Published:** 1982-09

**Authors:** M. R. Nowrousian, C. G. Schmidt

## Abstract

The effects of Cisplatin on marrow haemopoietic progenitor cells, WBC and RBC were measured and compared in F1 (CBA x C57BL) female mice. Dose/survival curves of Cisplatin for CFU-S, CFU-C and BFU-E were found to be simply exponential, indicating that the effect of the drug has no cell-cycle dependency. BFU-E also appeared significantly (P less than 0.001) more sensitive to Cisplatin than CFU-S and CFU-C. After a single dose of 12 mg/kg of Cisplatin, WBC, MNC and CFU-E were seen to be markedly less reduced and to recover much earlier than CFU-C, and particularly BFU-E and CFU-S. Results suggest that the drug is more toxic for earlier haemopoietic progenitor cells than for the more mature cells, and that the latter are not reliable parameters for complete haemopoietic recovery in mice after treatment with this agent. In the animals treated, there was also a subsequent significant decrease of the RBC count, accompanied by a marked increase of the marrow CFU-E concentration. Possible underlying mechanisms (e.g. alterations of RBC after exposure to Cisplatin) were discussed.


*Br. J. Cancer* (1982) **46**, 397

### EFFECTS OF CISPLATIN ON DIFFERENT HAEMOPOIETIC PROGENITOR CELLS IN MICE

M. R. NOWROUSIAN AND C. G. SCHMIDT

*From the West German Tumour Center, University of Essen, Department of Internal Medicine (Cancer Research), Essen, F.R.G.*

Received 5 October 1981 Accepted 29 April 1982

**Summary.—The effects of Cisplatin on marrow haemopoietic progenitor cells, WBC and RBC were measured and compared in $F_1$ (CBA × C57BL) female mice. Dose/survival curves of Cisplatin for CFU-S, CFU-C and BFU-E were found to be simply exponential, indicating that the effect of the drug has no cell-cycle dependency. BFU-E also appeared significantly ($P < 0{\cdot}001$) more sensitive to Cisplatin than CFU-S and CFU-C. After a single dose of 12 mg/kg of Cisplatin, WBC, MNC and CFU-E were seen to be markedly less reduced and to recover much earlier than CFU-C, and particularly BFU-E and CFU-S. Results suggest that the drug is more toxic for earlier haemopoietic progenitor cells than for the more mature cells, and that the latter are not reliable parameters for complete haemopoietic recovery in mice after treatment with this agent. In the animals treated, there was also a subsequent significant decrease of the RBC count, accompanied by a marked increase of the marrow CFU-E concentration. Possible underlying mechanisms (e.g. alterations of RBC after exposure to Cisplatin) were discussed.**

CISPLATIN (*cis*-diamminedichloroplatinum II) has been shown to be an effective antineoplastic agent in experimental animals and in man (Prestayko *et al.*, 1979). In earlier studies, the major dose-limiting factor in the clinical use of this agent was reported to be the dose-related and cumulative renal toxicity (Krakoff, 1979; Talley *et al.*, 1973). The myelotoxicity of Cisplatin became increasingly evident after high doses of the drug were given with nephrotoxicity reduced by hydration and diuretics (Prestayko *et al.*, 1979; Chary *et al.*, 1977). Recently, several investigators have reported severe myelosuppresion in patients treated with Cisplatin or chemotherapy combinations containing this agent (von Hoff *et al.*, 1979; Kuzur & Greco, 1980). The drug was found to induce not only leucocytopenia and thrombocytopenia, but also severe anaemia when given repeatedly, a phenomenon which is unusual in patient treated with combinations containing cytotoxic agents other than Cisplatin (Rossof *et al.*, 1972; von Hoff *et al.*, 1979; Kuzur & Greco, 1980). The anaemia was considered to be secondary to changes in erythropoiesis, and in some cases to haemolysis (Kuzur & Greco, 1980; Rothmann & Weick, 1981; Getaz *et al.*, 1980; Levi *et al.*, 1981; van Nguyen & Jaffe, 1981). However, the mechanisms responsible have not yet been thoroughly investigated. Moreover, few studies have been carried out to evaluate the effects of Cisplatin on haemopoiesis and particularly on haemopoietic stem cells. The drug has been shown to have a dose-related and cumulative toxicity for CFU-S and CFU-C in mice (Jenkins *et al.*, 1981; Dumenil *et al.*, in press) and to be more toxic to murine CFU-C than to human cells (Ogawa *et al.*, 1975).

The aim of the present study was to

Correspondence to: Dr M. R. Nowrousian, Westdeutsches Tumorzentrum, Innere Universitätsklinik und Poliklinik (Tumorforschung), Hufelandstrasse 55, 4300 Essen, F.R.G.

define the sensitivity of different haemopoietic stem cells to Cisplatin in mice, and to investigate the recovery of cells after a single dose of the agent equivalent in mice to doses usually given in man.

### MATERIALS AND METHODS

*Animals and drug administration.*—$F_1$ (CBA × C57BL) female mice, 10–12 weeks of age and weighing 20–25 g, were used, both for Cisplatin treatment and as recipients for CFU-S assays. Cisplatin (Haereus, Hanau, F.R.G.) was appropriately dissolved in 0·5 ml sterile water and given i.p. Survival of cells was determined 24 h after injection of various drug doses, and recovery of cells after a single dose of 12 mg/kg of Cisplatin. The latter lies between the $LD_{10}$ and the $LD_{50}$ for mice (Penta *et al.*, 1979) and using the conversion factor of Freireich *et al.* (1966) it is comparable to a single dose of 70 mg given to a human weighing 70 kg. Single-cell suspensions were prepared from flushed femoral marrow of 3 mice/group/point, and blood samples for individual WBC and RBC counts were obtained by severing the axillary vessels.

*CFU-S, CFU-C, BFU-E and CFU-E assays.*—CFU-S was assayed by the method of Till & McCulloch (1961). Recipient mice, at least 10 mice/point/group, were exposed to 7·5 Gy total-body irradiation (X-ray machine, rate 1·43 Gy/min, focal distance 40 cm) before injection of $4 \times 10^4$ nucleated cells from the marrow (MNC). Macroscopic surface colonies in the spleens were counted 9 days later. CFU-C were assayed according to the method of Bradley & Metcalf (1966) as modified by Iscove (1972). MNC $10^5$ were cultured in 1 ml α-medium containing 0·8% methylcellulose, 20% horse serum and 20% mouse heart-conditioned medium (Byrne *et al.*, 1978). BFU-E and CFU-E were assayed by the method of Iscove & Sieber (1975). MNC ($2 \times 10^5$) were cultured in 1 ml Iscove's modified Dulbecco's medium containing 0·8% methylcellulose, 1% bovine serum albumin, 15% foetal calf serum, 15% horse serum, $10^{-4}$M 2-mercaptoethanol and sheep-plasma erythropoietin (2 U for BFU-E, 0·2 U for CFU-E, Connaught). All cultures were set up in triplicates and incubated at 37°C in a humified atmosphere with 5% $CO_2$ in air. CFU-C were scored on Day 7, BFU-E on Day 9 and CFU-E on Day 2 of incubation. BFU-E and CFU-E colonies were stained using an improved benzidine staining technique (Gallicchio & Murphy, 1979).

*Statistical analysis.*—Linear regression was fitted according to the method of least squares. Significance of difference between the slopes of dose/survival curves was tested by comparing the regression coefficients using the *t* test. Recovery data were analysed on difference between the treated and control groups using both the *t* test and Mann–Whitney test.

### RESULTS

The marrow nucleated cell count decreased progressively up to 75% of the control values with increasing doses of Cisplatin between 0·5 and 3 mg/kg. Thereafter, no further significant reduction was observed with higher doses.

FIG. 1.—Dose/survival curves of CFU-C (■—■), CFU-S (●—.—●) and BFU-E (▲- - -▲) to Cisplatin in mice. Cell survival was determined 24 h after i.p. injection of the drug. Data represent the means ± s.e. from 3–8 separate experiments. The slope of the BFU-E curve (−0.41) differs significantly ($P < 0·001$) from those of CFU-C (−0·22) and CFU-S (−0·25).

FIG. 2.—Serial changes in RBC, WBC and MNC in mice after a single i.p. injection of 12 mg/kg of Cisplatin. Data were converted to % of control values. Vertical bars signify s.e. of 3–8 separate experiments. Shaded zones represent the means ± s.e. of all control values for RBC ($8.23 \pm 0{\cdot}1 \times 10^{12}$/l), WBC ($4983 \pm 574 \times 10^{9}$/l) and MNC ($11{\cdot}84 \pm 0{\cdot}16 \times 10^{6}$/femur) obtained in this study.

FIG. 3.—Recovery of marrow CFU-S and CFU-C in mice after a single i.p. injection of 12 mg/kg of Cisplatin. Data were converted to % of control values. Vertical bars signify s.e. of 3–8 separate experiments. Shaded zones represent the means ± s.e. of all control values for CFU-S (2809 ± 66/femur) and CFU-C (14622 ± 982/femur) obtained in this study.

The surviving fractions of CFU-S, CFU-C and BFU-E showed a continuous exponential decrease with increasing drug doses above 1 mg/kg (Fig. 1). BFU-E, in addition, appeared to be significantly ($P < 0{\cdot}001$) more sensitive to Cisplatin than CFU-S and CFU-C.

After a single dose of 12 mg/kg of Cisplatin, the WBC count showed a decrease up to 53% of the control values during the first 3 days, followed by a return to normal by Day 5 (Fig. 2). The RBC count remained unchanged for the first 2 days after drug application, but increased significantly ($P < 0{\cdot}05$) above normal levels by Days 3–5 and returned to normal by Day 8. During this period the animals frequently developed gastroenteritis and loss of weight. The increase of the RBC count might therefore be, at least partially, due to dehydration. From Day 10, the RBC number decreased slowly but significantly ($P < 0{\cdot}05$) below normal and reached 87% of the control values by Day 23. The cells, however, did not show any apparent changes in their size, shape or staining characteristics. The MNC had their nadir on Day 3 after treatment at 65% of the control values; the cell number returned to normal by Day 8.

The CFU-S number dropped rapidly to 5% of the control value on Day 1 after drug administration (Fig. 3). Thereafter, the CFU-S compartment size increased progressively to reach subnormal levels by Day 15, and remained subnormal as long as the cells were studied. The CFU-C were reduced up to 6% of the control value during the first 2 days after treatment; the cell concentration then started to rise, with a recovery of the cells being completed on Day 15.

The BFU-E had their nadir on Day 1 after treatment, where 0·85% of the control value was achieved (Fig. 4). Thereafter, the cells began to recover, but their

FIG. 4.—Recovery of marrow CFU-E and BFU-E in mice after a single i.p. injection of 12 mg/kg of Cisplatin. Data were converted to % of control values. Vertical bars signify the s.e. of 3–8 separate experiments. Shaded zones represent the means ± s.e. of all control values for CFU-E (10510 ± 414/femur) and BFU-E (1031 ± 37/femur) obtained in this study.

recovery was more delayed than that of CFU-S and CFU-C, since subnormal levels were not achieved before Day 17. The CFU-E showed the least degree of suppression and the most rapid recovery in comparison to the other cell types. From Day 10, concomitant with the decrease of the RBC count, the CFU-E number increased significantly ($P < 0{\cdot}05$) above normal levels and reached 167% of normal by Day 23.

### DISCUSSION

The exponential dose/survival curves for CFU-S, CFU-C and BFU-E indicate that the effect of Cisplatin has no cell-cycle dependency. This finding agrees with the observations suggesting a similar toxicity of Cisplatin for CFU-C both in normal and regenerating marrow and on cultured human lymphoma cells in different stages of the cell cycle (Ogawa *et al.*, 1975; Drewinko *et al.*, 1973). The differential sensitivity of the cells to Cisplatin has to be considered valid only for the experimental conditions studied, since the slopes of dose/survival curves could change with variations in the interval between the drug administration and the assay of stem cells (van Putten *et al.*, 1972). Additionally, the different response of BFU-E and CFU-C to Cisplatin might be due to the different maturation stages of these cells and it could be that earlier congeners of CFU-C were similarly sensitive as are BFU-E.

The more delayed recovery of CFU-S, BFU-E and CFU-C by comparison to MNC and WBC, suggest that the latter are not reliable parameters for complete haemopoietic recovery in mice after treatment with Cisplatin. If the same were true in humans, these indices, usually considered to be predictive for marrow toxicity of cytotoxic agents, could not be used for the evaluation of the true effect of Cisplatin on marrow function.

The recovery pattern of MNC and CFU-S after a single dose of 12 mg/kg of Cisplatin in mice is approximately comparable to that seen in the same animal species after exposure to 3–5 Gy whole-body irradiation (Valeriote *et al.*, 1968; Guzman & Lajtha, 1970). Additionally, the effects of Cisplatin on haemopoietic precursor cells resemble those of agents such as Busulphan and 1-bis(2-chloroethyl)-1-nitrosourea (BCNU), which have been shown to be more toxic to the earlier progenitor cells than to the more mature cells (Botnick *et al.*, 1981).

The toxicity of Cisplatin for CFU-S and CFU-C has been reported to be dose-related and cumulative (Jenkins *et al.*, 1981; Dumenil *et al.*, in press). Considering this and our data, similar toxicities were possible for BFU-E, and these toxic effects could increase more rapidly with higher drug doses than that for CFU-S and particularly CFU-C. The question therefore arises whether the pronounced Cisplatin toxicity for BFU-E could be the cause of the anaemia induced by this agent. Such a preferential BFU-E depression has been reported from a single patient studied (Rothmann & Weick,

1981). However, the haemopoietic effects of Cisplatin might be different in mouse and man, as reported from *in vitro* studies on CFU-C (Ogawa *et al.*, 1975). Further investigations are therefore needed to clarify the sensitivity of different haemopoetic stem cells to Cisplatin in man, and to evaluate the recovery of cells after exposure to repeated doses.

The more prominent anaemia could also be due to different *in vivo* maturation times of erythroid and granulocytic progenitor cells. For example, it might be that if both cell populations were suppressed equally by Cisplatin, the smaller number of cells involved in erythropoiesis per unit time would eventually lead to a more pronounced anaemia than granulocytopenia. In Cisplatin-treated mice, however, the anaemia was accompanied by a marked increase of marrow CFU-E, indicating an appropriate response of the erythropoietic system, despite the protracted BFU-E recovery. The latter might therefore be a result of the need for an enhanced production of mature cells, and is compatible with the observation made in anaemic mice, in which an increase of CFU-E was associated with a decrease of BFU-E (Hara & Ogawa, 1977). However, a delay in BFU-E recovery does not seem to be the cause of anaemia where normal or raised numbers of CFU-E are present. Although a high incidence of CFU-E has been shown to be not always reflected in effective production of mature cells (Testa, 1979; Peschle *et al.*, 1980), it also seems less likely that the reduced RBC count was a result of CFU-E maturation disturbances. Alternatively, the RBC number could be depressed by haemolysis. Haemolytic anaemia has been reported in patients treated repeatedly with Cisplatin (Getaz *et al.*, 1980; Levi *et al.*, 1981; van Nguyen & Jaffe, 1981). The haemolysis was suggested as induced by antibodies reacting with Cisplatin–RBC-membrane complexes (Getaz *et al.*, 1980). Such an antibody-mediated haemolysis seems unlikely in our experiments, because of the single dose of the agent given, but other mechanisms (*e.g.* toxic effects of Cisplatin on RBC themselves) would also be able to induce a shortened cell survival, leading to anaemia. Therefore, appropriate studies concerning possible alterations of RBC after exposure to Cisplatin could be of value.

This work was supported by a grant from the Bundesministerium fuer Forschung und Technologie in Bonn.

### REFERENCES

Botnick, L. E., Hannon, E. C., Vigneulle, R. & Hellman, S. (1981) Differential effects of cytotoxic agents on haematopoietic progenitors. *Cancer Res.*, **41**, 2338.

Bradley, T. R., & Metcalf, D. (1966) The growth of mouse bone marrow cells *in vitro*. *Aust. J. exp. Biol. Med. Sci.*, **44**, 287.

Byrne, P. V., Heit, W. & Kubanek, B. (1978) Stimulation of *in vitro* granulocyte macrophage colony formation by mouse heart conditioned medium. *Br. J. Haematol.*, **40**, 197.

Chary, K. K., Higby, D. J., Henderson, E. S. & Swinerton, K. D. (1977) Phase 1 study of high dose *cis*-dichlorodiammineplatinum (II) with forced diuresis. *Cancer Treat. Rep.*, **61**, 367.

Drewinko, B., Brown, B. W. & Gottlieb, J. A. (1973) The effect of *cis*-diamminedichloroplatinum (II) on cultured human lymphoma cells and its therapeutic implication. *Cancer Res.*, **33**, 3091.

Dumenil, D., Droz, D., Droz, J. P. & Frindel, E. (1982) Some effects of chemotherapeutic drugs. III. Short and long term effects of cisplatinum on various hematopoietic compartments and on kidney of the mouse. *Cancer Chemother. Pharmacol.* (in press).

Freireich, D. J., Gehan, E. A., Rall, D. P., Schmidt, L. A. & Skipper, H. E. (1966) Quantitative comparison of toxicity of anticancer agents in mouse, rat, hamster, dog, monkey and man. *Cancer Chemother. Rep.*, **50**, 219.

Gallicchio, V. S. & Murphy, J., Jr (1979) Erythropoiesis *in vitro*. II. Cytochemical enumeration of erythroid stem cells (CFU-E and BFU-E) from normal mouse and human hematopoietic tissues. *Exp. Hematol.*, **7**, 219.

Getaz, E. P., Beckly, S., Fitzpatrick, J. & Dozier, A. (1980) *Cis*-platin-induced haemolysis. *N. Engl. J. Med.*, **302**, 334.

Guzman, E. & Lajtha, L. G. (1970) Some comparisons of the kinetic properties of femoral and splenic haematopoietic stem cells. *Cell Tissue Kinet.*, **3**, 91.

Hara, H. & Ogawa, M. (1977) Erythropoietic precursors in mice under erythropoietic stimulation and suppression. *Exp. Hematol.*, **5**, 141.

Iscove, N. N. (1972) Technique for culture of human CFU-C. In In Vitro *Culture of Hemopoietic Cells* (Ed. van Bekkum & Dicke). Rijswijk: Radiobiological Institute. p. 459.

Iscove, N. N. & Sieber, F. (1975) Erythroid progenitors in mouse bone marrow detected by macroscopic colony formation in culture. *Exp Hematol.*, **3**, 32.

Jenkins, V. K., Perry, R. R. & Goodrich, W. E (1981) Effects of *cis*-diamminedichloroplatinum

(II) on hematopoietic stem cells in mice. *Exp. Hematol.*, **9**, 281.

KRAKOFF, I. H. (1979) Nephrotoxicity of *cis*-diamminedichloroplatinum (II). *Cancer Treat., Rep.*, **63**, 1523.

KUZUR, M. E. & GRECO, F. A. (1980) Cisplatin-induced anaemia. *N. Engl. J. Med.*, **302**, 110.

LEVI, A., ARONEY, R. S. & DALLEY, D. N. (1981) Haemolytic anaemia after cisplatin treatment. *Br. Med. J.*, **282**, 2003.

OGAWA, M., GALE, G. R. & KLEIN, S. S. (1975) Effects of *cis*-diamminedichloroplatinum (NSC 119875) on murine and human hematopoietic precursor cells. *Cancer Res.*, **35**, 1398.

PENTA, L. S., ROZENCWEIG, M., GUARINE, A. M. & MUGGIA, F. M. (1979) Mouse and large-animal toxicology studies of twelve antitumor agents: Relevance to starting dose for phase I clinical trials, *Cancer Chemother. Pharmacol.*, **3**, 97.

PESCHLE, C., MIGLIACCIO, G., LETTIERI, F., & 5 others (1980) Kinetics of erythroid precursors in mice infected with the anemic or the polycythemic strain of Friend leukemia virus. *Proc. Natl Acad. Sci.*, **77**, 2054.

PRESTAYKO, A. W., D'AOUST, J. C., ISSELL, B. F. & GROOKE, S. T. (1979) Cisplatin (*cis*-diammine-dichloroplatinum II). *Cancer Treatment Rev*, **6**, 17.

ROSSOF, A. H., SLAYTON, R. E. & PERLIA, C. P. (1972) Preliminary clinical experience with *cis*-diamminedichloroplatinum (II) (NSCI 19875, CACP). *Cancer*, **30**, 1451.

ROTHMANN, S. A. & WEICK, J. K. (1981) Cisplatin toxicity for erythroid precursors. *N. Engl. J. Med.*, **304**, 360.

TALLEY, R. W., O'BRIEN, R. M., GUTTERMANN, J., BROWNLEE, R. W. & MCCREDIE, K. B. (1973) Clinical evaluation of toxic effects of *cis*-diammin-edichloroplatinum (NSC 119875)—phase 1 clinical study. *Cancer Chemother. Rep.*, **57**, 465.

TESTA, N. G. (1979) Erythroid progenitor cells: Their relevance for the study of haematological disease. *Clin. Haematol.*, **8**, 311.

TILL, J. E. & MCCULLOCH, E. A. (1961) A direct measurement of the radiation sensitivity of normal mouse bone marrow cells. *Radiat. Res.*, **14**, 213.

VALERIOTE, F. A., COLLINS, D. C. & BRUCE, W. R. (1968) Hematological recovery in the mouse following single doses of gamma radiation and cyclophosphamide. *Radiat. Res.*, **33**, 501.

VAN NGUYEN, B. & JAFFE, N. (1981) Cisplatin-induced anaemia. *Cancer Treat. Rep.*, **65**, 1121.

VAN PUTTEN, L. M., LELIEVELD, P. & KRAM-IDSENGA, L. K. (1972) Cell-cycle specificity and therapeutic effectiveness of cytotoxic agents. *Cancer Chemother. Rep.*, **56**, 691.

VON HOFF, D. D., SCHILSKY, R., REICHERT, C. M. & 4 others (1979) Toxic effects of *cis*-diamminedi-chloroplatinum (II) in man. *Cancer Treat. Rep.*, **63**, 1527.


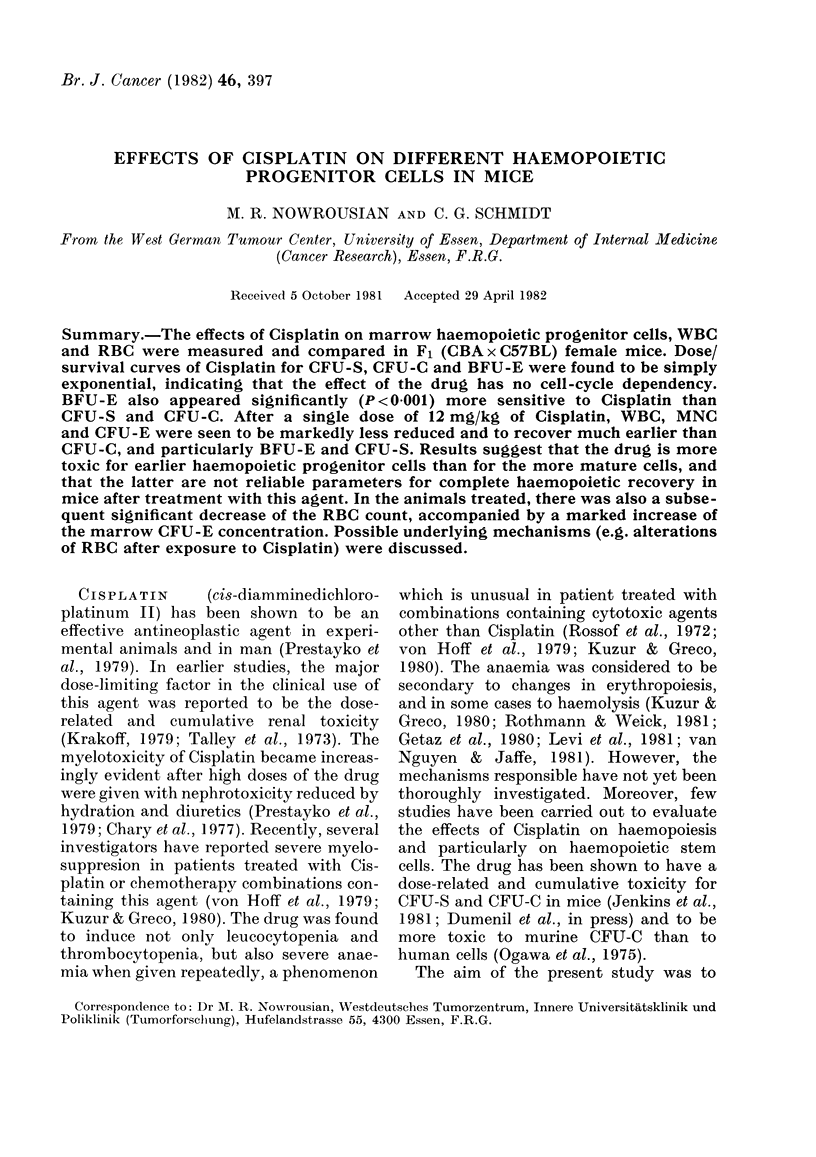

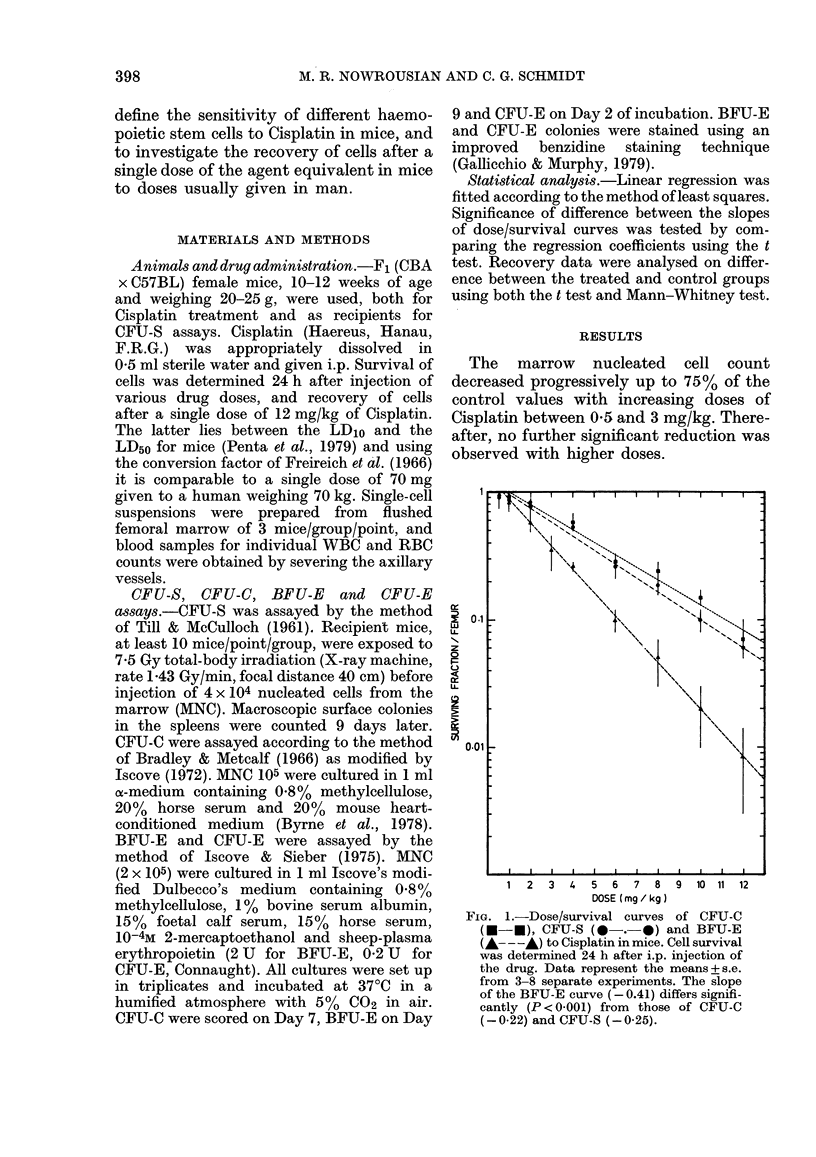

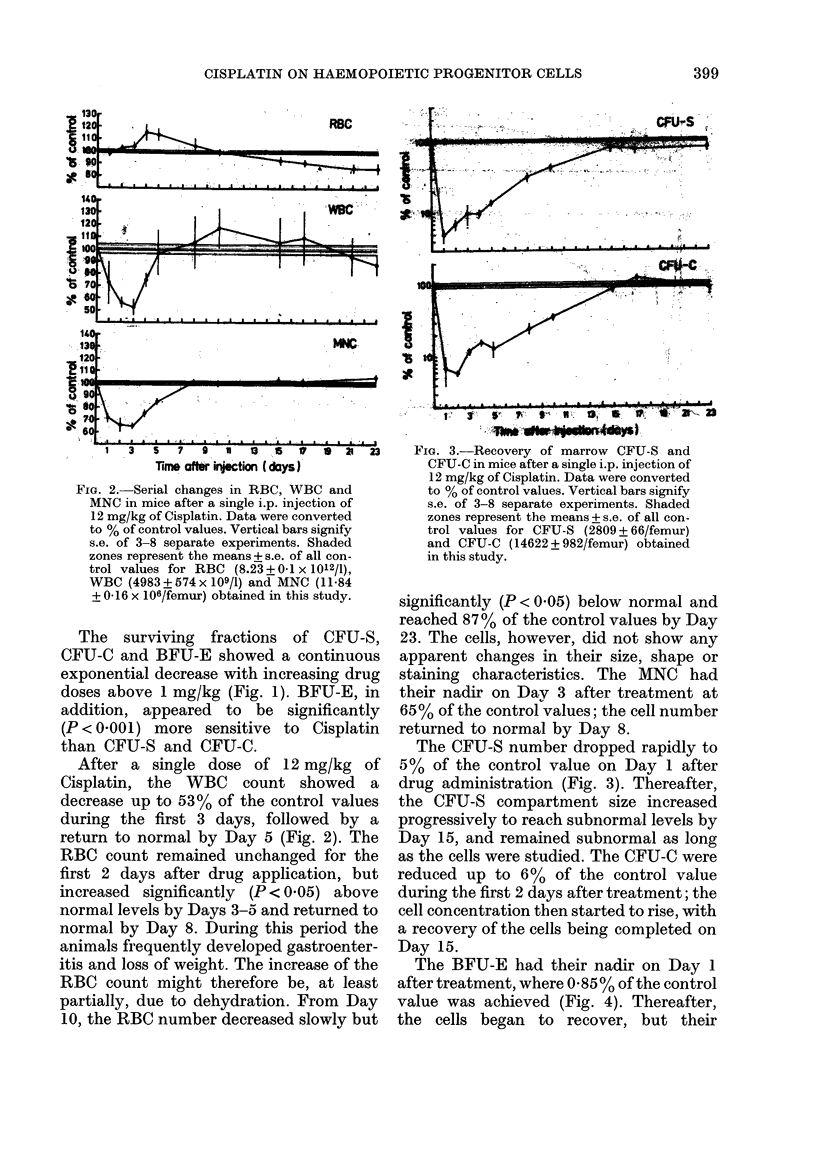

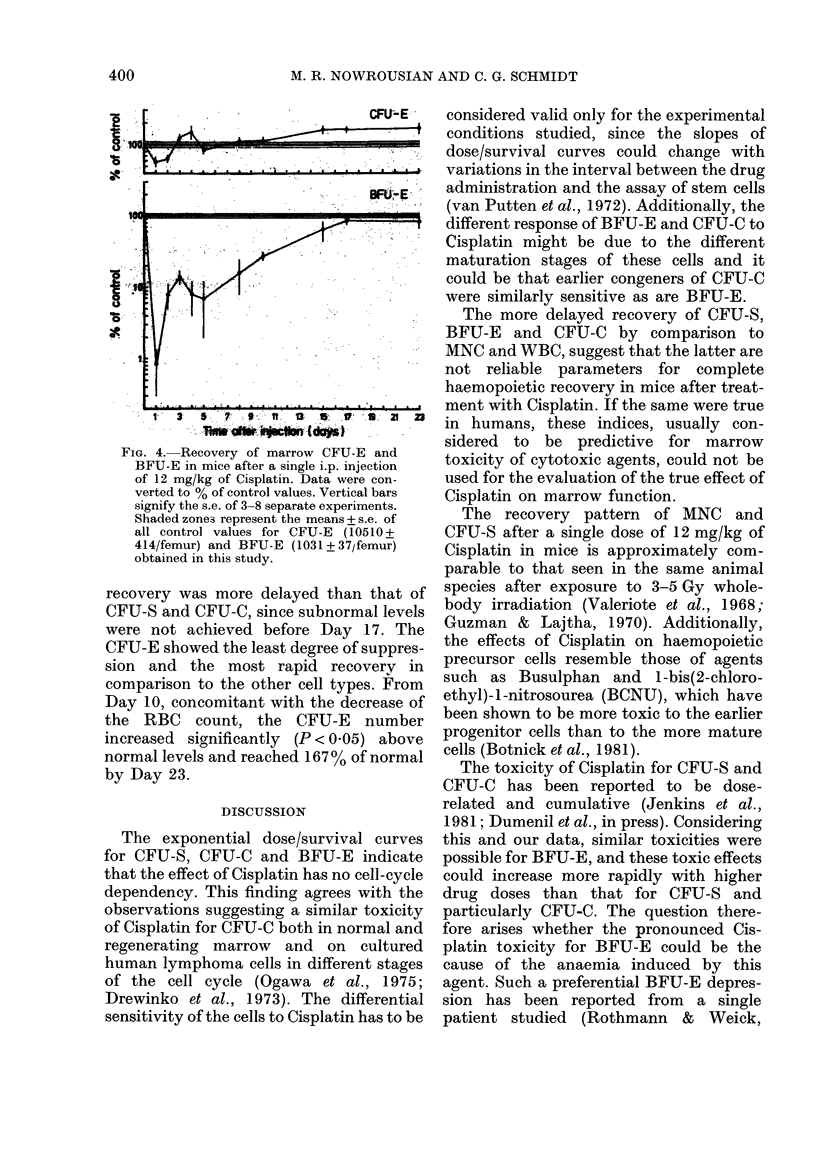

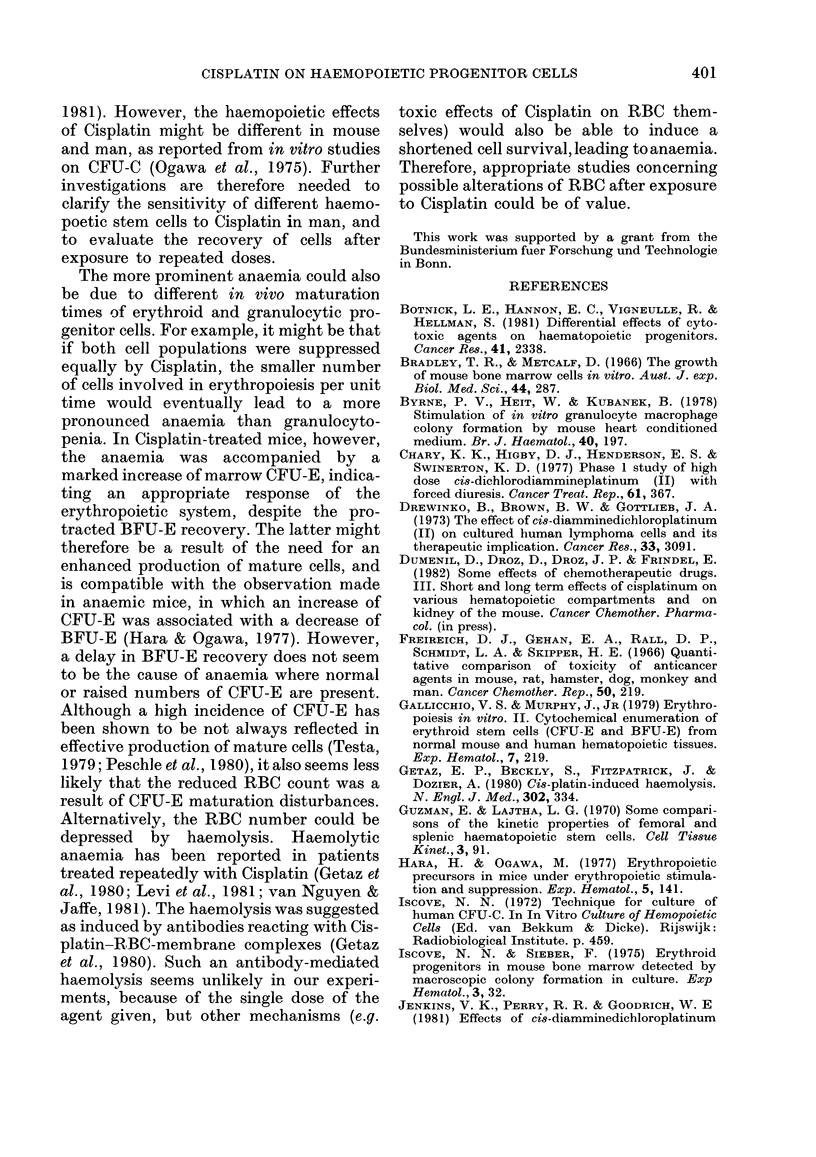

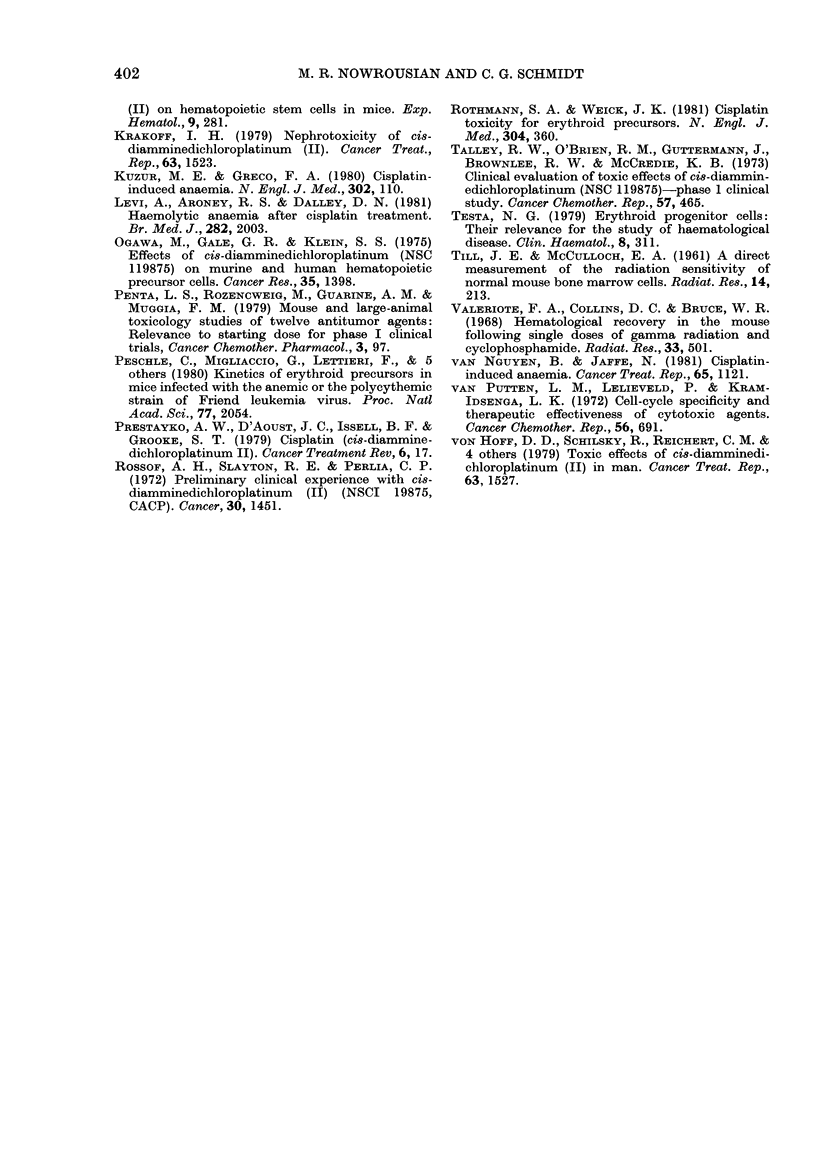


## References

[OCR_00434] Botnick L. E., Hannon E. C., Vigneulle R., Hellman S. (1981). Differential effects of cytotoxic agents on hematopoietic progenitors.. Cancer Res.

[OCR_00440] Bradley T. R., Metcalf D. (1966). The growth of mouse bone marrow cells in vitro.. Aust J Exp Biol Med Sci.

[OCR_00445] Byrne P. V., Heit W., Kubanek B. (1978). Stimulation of in vitro granulocyte--macrophage colony formation by mouse heart conditioned medium.. Br J Haematol.

[OCR_00451] Chary K. K., Higby D. J., Henderson E. S., Swinerton K. D. (1977). Phase I study of high-dose cis-dichlorodiammineplatinum(II) with forced diuresis.. Cancer Treat Rep.

[OCR_00457] Drewinko B., Brown B. W., Gottlieb J. A. (1973). The effect of cis-diamminedichloroplatinum (II) on cultured human lymphoma cells and its therapeutic implications.. Cancer Res.

[OCR_00471] Freireich E. J., Gehan E. A., Rall D. P., Schmidt L. H., Skipper H. E. (1966). Quantitative comparison of toxicity of anticancer agents in mouse, rat, hamster, dog, monkey, and man.. Cancer Chemother Rep.

[OCR_00478] Gallicchio V. S., Murphy M. J. (1979). In vitro erythropoiesis. II. Cytochemical enumeration of erythroid stem cells (CFU-e and BFU-e) from normal mouse and human hematopoietic tissues.. Exp Hematol.

[OCR_00485] Getaz E. P., Beckley S., Fitzpatrick J., Dozier A. (1980). Cisplatin-induced hemolysis.. N Engl J Med.

[OCR_00490] Guzman E., Lajtha L. G. (1970). Some comparisons of the kinetic properties of femoral and splenic haemopoietic stem cells.. Cell Tissue Kinet.

[OCR_00496] Hara H., Ogawa M. (1977). Erythropoietic precursors in mice under erythropoietic stimulation and suppression.. Exp Hematol.

[OCR_00507] Iscove N. N., Sieber F. (1975). Erythroid progenitors in mouse bone marrow detected by macroscopic colony formation in culture.. Exp Hematol.

[OCR_00513] Jenkins V. K., Perry R. R., Goodrich W. E. (1981). Effects of Cis-diamminedichloroplatinum (II) on hematopoietic stem cells in mice.. Exp Hematol.

[OCR_00522] Krakoff I. H. (1979). Nephrotoxicity of cis-dichlorodiammineplatinum(II).. Cancer Treat Rep.

[OCR_00527] Kuzur M. E., Greco F. A. (1980). Cisplatin-induced anemia.. N Engl J Med.

[OCR_00531] Levi J. A., Aroney R. S., Dalley D. N. (1981). Haemolytic anaemia after cisplatin treatment.. Br Med J (Clin Res Ed).

[OCR_00595] Nguyen B. V., Jaffe N., Lichtiger B. (1981). Cisplatin-induced anemia.. Cancer Treat Rep.

[OCR_00536] Ogawa M., Gale G. R., Keirn S. S. (1975). Effects of cis-diamminedichloroplatinum (NSC 119875) on murine and human hemopoietic precursor cells.. Cancer Res.

[OCR_00542] Penta J. S., Rozencweig M., Guarino A. M., Muggia F. M. (1979). Mouse and large-animal toxicology studies of twelve antitumor agents: relevance to starting dose for phase I clinical trials.. Cancer Chemother Pharmacol.

[OCR_00549] Peschle C., Migliaccio G., Lettieri F., Migliaccio A. R., Ceccarelli R., Barba P., Titti F., Rossi G. B. (1980). Kinetics of erythroid precursors in mice infected with the anemic or the polycythemic strain of Friend leukemia virus.. Proc Natl Acad Sci U S A.

[OCR_00560] Rossof A. H., Slayton R. E., Perlia C. P. (1972). Preliminary clinical experience with cis-diamminedichloroplatinum (II) (NSC 119875, CACP).. Cancer.

[OCR_00566] Rothmann S. A., Weick J. K. (1981). Cisplatin toxicity for erythroid precursors.. N Engl J Med.

[OCR_00583] TILL J. E., McCULLOCH E. A. (1961). A direct measurement of the radiation sensitivity of normal mouse bone marrow cells.. Radiat Res.

[OCR_00571] Talley R. W., O'Bryan R. M., Gutterman J. U., Brownlee R. W., McCredie K. B. (1973). Clinical evaluation of toxic effects of cis-diamminedichloroplatinum (NSC-119875)--phase I clinical study.. Cancer Chemother Rep.

[OCR_00578] Testa N. G. (1979). Erythroid progenitor cells: their relevance for the study of haematological disease.. Clin Haematol.

[OCR_00589] Valeriote F. A., Collins D. C., Bruce W. R. (1968). Hematological recovery in the mouse following single doses of gamma radiation and cyclophosphamide.. Radiat Res.

[OCR_00601] Van Putten L. M., Lelieveld P., Kram-Idsenga L. K. (1972). Cell-cycle specificity and therapeutic effectiveness of cytostatic agents.. Cancer Chemother Rep.

[OCR_00605] Von Hoff D. D., Schilsky R., Reichert C. M., Reddick R. L., Rozencweig M., Young R. C., Muggia F. M. (1979). Toxic effects of cis-dichlorodiammineplatinum(II) in man.. Cancer Treat Rep.

